# Fuzzy Neural Network PID-Based Constant Deceleration Control for Automated Mine Electric Vehicles Using EMB System

**DOI:** 10.3390/s24072129

**Published:** 2024-03-27

**Authors:** Jian Li, Chi Ma, Yuqiang Jiang

**Affiliations:** School of Mechanical and Electrical Engineering, China University of Mining and Technology, Xuzhou 221116, China; lijian0609seu@163.com (J.L.); machicumt@163.com (C.M.)

**Keywords:** underground electric trackless rubber-tired vehicle, autonomous driving, constant deceleration control, fuzzy neural network PID algorithm, electro-mechanical brake system, intelligent chassis control

## Abstract

It is urgent for automated electric transportation vehicles in coal mines to have the ability of self-adaptive tracking target constant deceleration to ensure stable and safe braking effects in long underground roadways. However, the current braking control system of underground electric trackless rubber-tired vehicles (UETRVs) still adopts multi-level constant braking torque control, which cannot achieve target deceleration closed-loop control. To overcome the disadvantages of lower safety and comfort, and the non-precise stopping distance, this article describes the architecture and working principle of constant deceleration braking systems with an electro-mechanical braking actuator. Then, a deceleration closed-loop control algorithm based on fuzzy neural network PID is proposed and simulated in Matlab/Simulink. Finally, an actual brake control unit (BCU) is built and tested in a real industrial field setting. The test illustrates the feasibility of this constant deceleration control algorithm, which can achieve constant decelerations within a very short time and maintain a constant value of ${-2.5\;m/s}^{2}$ within a deviation of $\pm 0.1\;m/s^{2}$, compared with the deviation of $0.11\;m/s^{2}$ of fuzzy PID and the deviation of $0.13\;m/s^{2}$ of classic PID. This BCU can provide electric and automated mine vehicles with active and smooth deceleration performance, which improves the level of electrification and automation for mine transport machinery.

## 1. Introduction

An underground electric trackless rubber-tired vehicle (UETRV) is the key transportation equipment connecting ground and underground facilities during the process of mine production and transportation. The braking system is the key device to ensure the safe operation of UETRVs, especially in emergencies, and should allow timely and smooth parking, in order to avoid further accidents. With the increase in mining depth and slope length, higher requirements are put forward for the stability of continuous braking. Active constant deceleration control is a necessary technology to ensure the safety of UETRV braking, which is also an indispensable technology for UETRV and can further promote the realization of unmanned mine transportation. 

Currently, the braking force of UETRV is calculated according to the brake signal from the brake pedal, and then applied to the brake disc by the brake actuator. In fact, the dynamics of UETRV are related to a variety of vehicle state parameters, including road ramps, vehicle loads, varying friction coefficients, and sensor errors; therefore, the fixed target braking force cannot guarantee actual deceleration stability in tracking the ideal target deceleration [1]. Therefore, the braking control method based on single-force information feedback cannot achieve stable control of deceleration even when vehicle and road parameters are fixed [2]. 

In comparison with a constant braking torque, a constant deceleration braking system based on real-time deceleration feedback information control can adaptively adjust the output of the braking torque, which aims at achieving nearly constant braking deceleration. A few scholars have placed focus on this active braking system. In [3], a fuzzy PID controller of a constant deceleration braking control strategy for the disc brake of ultra-deep mine hoists was designed, and its performance was simulated based on co-simulation with Adams v.2016 and Simulink v.2016. The simulation results show that there is an inevitable small jitter in the deceleration speed. Ma et al. studied [4] a constant deceleration compensation device for a mine hoist that combines fuzzy logic with neural networks to adjust PID parameters and that was simulated based on co-simulation with AMESim v.2016 and Simulink. 

Although there are a few studies working on the constant deceleration braking mechanism in mine machinery, some research contributions in the field of railway vehicles and urban trains can be referenced. Wang and Wen et al. [5] introduce the idea that the pressure of a brake cylinder in an urban train can be adjusted adaptively to track target deceleration based on the deceleration signal from the deceleration sensor and the dynamic friction coefficient derived from machine learning method; the experiments show good deceleration tracking ability. Aiming at improving the deficiency of the non-deceleration control mode and the uncertainty of multi-parameters, a new deceleration control algorithm based on unified parameter estimation is presented in [6], the test results of which show that the average deceleration is relatively stable and the instantaneous deceleration error is small under the disturbance of the uncertain friction coefficient, road slope, vehicle load, and braking force feedback error. In [7], the variation in friction coefficients between the disc and gasket is considered when analyzing the braking performance of a rail vehicle. A multi-parameter mathematical formula including thermal effect is presented to describe the friction characteristics, and the influence of the variable friction coefficient on actual braking force control is proven via hardware-in-the-loop simulation (HILS). In [8], the pneumatic valve of a train pneumatic braking system is calculated via sliding mode control, and the wheel anti-skid control of the rail vehicle pneumatic braking system is designed and verified in HILS. In [9], for solving the wheel skidding problem of electric vehicles during acceleration when a vehicle starts, a sliding mode control method for acceleration and slip rate is designed. Considering the oscillation of the system, a fuzzy controller is used to adjust the switching function parameters of the slide mode controller. 

There are a few algorithms for constant deceleration and speed control that can give us more novel ideas in the field of advanced driving assistant systems (ADASs) and automated vehicles. In [10], based on the physical equations of an automobile, the longitudinal model of the automobile is designed and used to compensate for the nonlinear dynamic behavior in the control loop, and the parameters of the model are estimated by using the data-based identification technique. For maintaining a stable speed on a road with variable slopes, Ref. [11] designed a preview speed control algorithm by integrating future slope and target speed into an augmented optimal control problem to achieve a smooth speed tracking effect with small computation costs for connected automated vehicles (CAVs). 

In addition, a few optimized PID controllers based on intelligent algorithms are also proposed in industry control. A Sigmoid-PID (SPID) controller is designed in [12] to make the automatic voltage regulator of the synchronous generator be controlled by the PID controller to reach the rated voltage more quickly with a shorter-voltage steady-state error. The authors in [13] proposed a new intelligent controller based on brain emotion learning (BELBIC) to solve the problems of vehicle path tracking and collision control in high-speed automatic highway systems, where an improved BELBIC controller is applied to the sixth-order vehicle model, which can track any normal path in the simulation platform. Ref. [14] designed a Sigmoid-based neuroendocrine PID controller for crane systems, whose main advantage is that the hormone secretion rate of neuroendocrine-PID can be varied according to the change in error. The simulation test proves that this improved neuroendocrine-PID provides better control performances in terms of the objective function, the total norm of error, and the total norm of input compared to PID and neuroendocrine-PID controllers. In [15], genetic algorithm is used to determine the composition and parameters of each meta-heuristic and the order of mixed meta-heuristics. And the hybrid heuristics for an aircraft landing problem and two-dimensional assembly optimization problem are designed automatically and evaluated by using genetic algorithm. However, the design of mixed meta-heuristics is time-consuming and requires expert knowledge of the different mixed meta-heuristics. 

For solving problems of pressure fluctuation and low accuracy of deceleration control when Electric Stable System (ESC) is used as braking actuator of Automated Cruise Control (ACC) system, a hierarchical adaptive cruise control structure is proposed in [16], the lower layer of which is a deceleration controller based on nonlinear model predictive control (MPC); the simulation results show that compared with the traditional PID deceleration controller, the deceleration control accuracy is effectively improved. In [17], a composite longitudinal control strategy is designed, which combines a steady-state controller with an auto-disturbance rejection state feedback controller for speed control during acceleration and deceleration. Finally, the stability of the velocity error is analyzed with the global sector condition. In [18], the deceleration control is transformed into the hydraulic control of the main cylinder; a sliding mode controller is designed to realize the pressure control when dealing with uncertainty between cylinder displacement and hydraulic pressure, where the hydraulic reference value is calculated by feedforward–feedback method to form a deceleration closed loop. Ref. [19] introduces the longitudinal acceleration test of a city bus during rapid acceleration and braking. The results show that the road condition and the type of road surface have a significant influence on the longitudinal acceleration; in particular, the maximum deceleration recorded in the urban bus and bus test is much lower than that recorded in a sudden braking maneuver. Ref. [20] proposed a hierarchical controller to actively control deceleration of a pure electric and autonomous vehicle, whose lower controller can track the hydraulic pressure of the wheel cylinder with closed-loop pressure feedback, and the upper controller determines the target cylinder braking pressure corresponding to vehicle dynamic equations. 

The dynamic parameters of a vehicle can importantly affect the motion performance of the vehicle. In [21], a second-order linear expansion state observer-based method for estimating the friction coefficient of the road surface considering load transfer between front and rear axles is proposed. Simulation results show that the observer can estimate the road friction coefficient in real time and accurately under different road conditions. In [22], based on the real-time and accurate torque and speed information of a four-wheel electric vehicle and Lyapunov stability theory, a slip-based algorithm for estimating the maximum road friction coefficient is designed. Through Lyapunov stability analysis, the convergence of the estimated errors of the longitudinal force and maximum pavement friction coefficient is proved. Ref. [23] introduces the design of an acceleration measurement system for measuring the three-axis acceleration of an automobile by using the MEMS accelerometer, which quantizes the output of the accelerometer by using the ARM microcontroller and suppresses the random noise generated during the measurement with a Kalman filter. In [24], FFNN is used to learn the relationship between vehicle mass and other state parameters. In the dynamics-based approach, the forgetting factor recursive least squares (RLS) based on the vehicle dynamics model is used to estimate the vehicle mass. Fuzzy logic is used to fuse the two methods. Simulation results show that the fusion method has better stability and robustness. Ref. [25] proposed a kind of real-time estimation of road slope based on a recursive Kalman filter, which takes into account road slope, tire rolling friction coefficient, and air resistance coefficient. In [26], Hybrid Extended Kalman filter (EKF) and recursive least squares (RLS) algorithms are used to estimate road gradients and vehicle masses, which are adopted to calculate total target braking torque and can effectively improve braking performance and the recovery energy. 

In real conditions, it is very difficult to accurately estimate parameters such as tire–road friction coefficient, road slope, dynamic mass, etc. [27,28]; meanwhile, the fuzzy algorithm and neural network control have advantages of adaptive parameter adjustment [29,30,31,32]. Therefore, this paper combined fuzzy control and neural network algorithm into a classic PID architecture to achieve closed-loop deceleration control. 

The main contributions of this paper are listed as follows:

- A constant deceleration control architecture based on a dynamic model of an electro-mechanical braking actuator is proposed, which utilized a deceleration sensor to conduct a closed-loop deceleration control system.
- A fuzzy neural network (FNN) deceleration control algorithm is proposed, where the fuzzy neural network unit can adaptively calculate the changing value to adjust gain parameters $K_{p},\;K_{i},{\;K}_{d}$ of the PID controller.
- The deceleration curve is proved by simulation to be relatively smoother in the normal braking process.
- A practical ECU with explosion-proof processing for deceleration braking control is developed and tested in a real UETRV on a test road, which can prove the stable deceleration rate performance of our proposed control strategy.

The rest of this paper is described as follows. The next section introduces the principle of the braking system, proposed deceleration closed-loop control architecture, and a simple dynamic model of the EMB system referenced in previous works [33,34] for this UETRV. Section 3 presents the fuzzy neural network with stretching factor-based vehicle deceleration rate controller, which can adaptively tune parameters of three gain values of PID in real time. The weight matrix parameters of the fuzzy neural network is also trained in this section. The results and analysis of simulations based on Simulink are shown in Section 4. Vehicle experiments on a test road are presented and discussed in Section 5. Finally, Section 6 describes some conclusions. 

## 2. The Architecture and Working Principle of Active Deceleration Control Systems in UETRV

### 2.1. Active Braking System Architecture with EMB Actuator

It is known that an EMB system has faster force response speed than hydraulic braking [35] and it is easy to construct a distributed braking system, which can separately adjust the actual braking force of every wheel [36,37]. Therefore, for enhancing the response performance of constant acceleration tracking control, an active braking system based on an EMB actuator in previous work is designed. 

**Figure 1:**
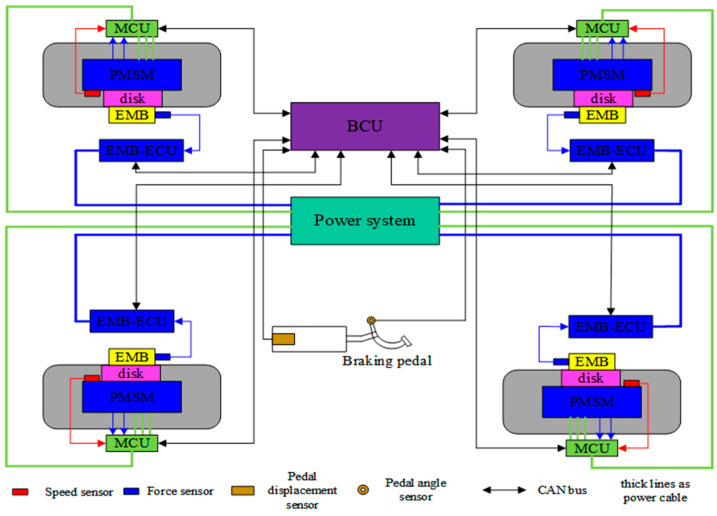
Architecture of distributed EMB system.

In Figure 1, every wheel of the UETRV is equipped with a single EMB actuator controlled by its EMB-ECU. Firstly, the braking control unit (BCU) calculates the overall braking force remand based on pedal input and further calculates the target braking force of every vehicle wheel, which will be sent into every EMB-ECU. Hence, every EMB-ECU can separately adjust the output of the brake clamping force via receiving a clamping force command from the braking control unit (BCU). The overall distributed EMB system transmits the control signal by Controller Area Network (CAN), which is a very reliable and popular communication technology for electric automotive systems. 

### 2.2. Simple Model of Single EMB Actuator 

In this paper, the proposed constant deceleration braking system is integrated with an EMB actuator, which means a real BBW system with active deceleration control. Hence, this section introduces a simple EMB model, which consists of a planetary reducer, a ball screw, frictional pads, and a braking disc [38].

#### 2.2.1. Model of Torque Motor

The SPMSM is selected as the torque motor in this EMB actuator. According to the working principle of PMSM, the SPMSM torque equation can be show as Equation (1).

(1) $$T_{m}=\frac{3}{2}n_{p}[\phi i_{q}+(L_{d}-L_{q})i_{d}i_{q}]$$

If *i_d_* is fixed at zero, the torque will go up to the maximum value. Hence, Equation (1) can be further simplified to a form similar to the DC motor torque formula, in which current can have a linear relationship with torque:

(2) $$T_{m}=\frac{3}{2}n_{p}\phi i_{q}$$

where the torque constant *K_t_* further replaces $\frac{3}{2}n_{p}\phi$. Therefore, Equation (2) can be simplified again as follows:

(3) $$T_{m}=K_{t}i$$

#### 2.2.2. Modeling of Mechanical Components

(1) Transmission mechanism

A reduction gear is utilized to enlarge the force generated from the motor. Subsequently, a ball screw can generate linear motion of a ball screw nut, which directly generates clamping force on the pad. The load torque $T_{L}$ is generated from the clamping force between pads and disc, and can be calculated as follows:

(4) $$T_{L}=\frac{1}{2\pi }\frac{L_{s}}{\eta_{g}n_{g}}\frac{1}{\eta_{s}}F_{cl}$$

where $\eta_{g}$ represents the efficiency coefficient of the gearbox and $\eta_{s}$ represents the efficiency coefficient of the ball screw; $n_{g}$ is the reduction ratio of the gear, $L_{s}$ is the lead of the ball screw. Equation (4) is usually expressed as $T_{L}=k_{cl}F_{cl}$, in which $k_{cl}$ is the total gearing transfer gain. 

$T_{m}$ should be specifically expressed as Equation (5) for further controller design:

(5) $$T_{m}=J_{m}\frac{d\omega_{m}}{dt}+B_{f}\omega_{m}+T_{L}$$

where $J_{m}$ is the rotor inertia constant, and $B_{f}$ is the damping coefficient of the rotor.

(2) Load model

The positive axial clamping force of the brake pads on the brake disc produces the friction torque required for braking, while the axial reaction of the lead screw on the motor and the reducer forms the load torque of the brake motor. This clamping force directly produces the incremental displacement of the screw head under disc contact, or the axial deformation of the brake disc, and the clamping force and the lead screw head displacement can be fitted using the following third-order polynomials, given as Equation (6):

(6) $$F_{cl}=A_{1}s^{3}+A_{2}s^{2}+A_{3}s$$

where $A_{1},A_{2},A_{3}$ are the fitting coefficients of the controlled clamping force; s is the displacement of the shape of the friction disc and also the displacement of the screw heads, mm.

(3) Braking disc model

The head of the ball screw pushes the pads into contact and clamps the brake disc, where the two sides of the brake disc are applied with equivalent friction torque. So the friction torque of a single EMB is given as:

(7) $$T_{\mu }=2F_{cl}\cdot R_{b}\cdot \mu_{b}$$

where $F_{cl}$ is the clamping force, N; $\mu_{b}$ is the friction coefficient of the friction plate; $R_{b}$ is the effective radius of the brake disc, m. 

**Figure 2:**
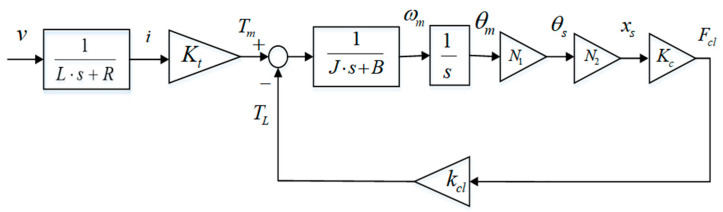
EMB actuator model.

Based on the above contents, a mathematical model of the EMB actuator is described in Figure 2. The relationship between current and voltage is simplified as a one-order transfer function with parameters L and R. The ball screw angle $\theta_{s}$ and its displacement $x_{s}$ have a proportional relationship, expressed as $x_{s}=N_{2}\theta_{s}.N_{2}=\frac{L}{2},\mathrm{where}\;L$ is lead of the ball screw. 

Figure 2 shows the simple EMB actuator model, which really generates actual braking clamping to achieve friction torque between the disc and pads. The force closed-loop controller utilized to track target clamping force in this constant deceleration braking system is a cascaded PID architecture, which will be introduced in Section 3 in detail. In Figure 2, the total equation from input voltage v to output clamping force $F_{cl}$ is described as follows:

(8) $$F_{cl}=\frac{\left(K_{t}N_{1}N_{2}K_{c})/[(L\cdot s+R)(J\cdot s+B)s\right]}{\left(1+\frac{N_{1}N_{2}K_{c}k_{cl}}{(J\cdot s+B)\cdot s}\right)}v$$

The real clamping force can be tracked by controlling the current of the torque motor, which can maintain the UETRV in keeping a smooth deceleration under the control of our proposed fuzzy neural network in the braking process. 

### 2.3. Principle of Active Deceleration Control for EMB System 

**Figure 3:**
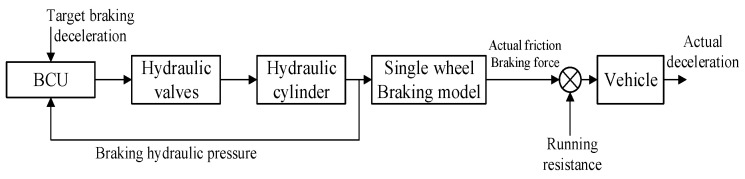
Conventional non-deceleration control mode.

Under the conventional braking technology, as shown in Figure 3, the BCU of the hydraulic brake system only calculates the target braking force based on the brake pedal signal and further converts it into the target pressure of the hydraulic cylinder, and the classical PID controller is used to realize the closed-loop tracking of the target hydraulic pressure. It should be pointed out that since the constant deceleration control system proposed in this paper is based on the EMB system, the target clamping force of the EMB actuator which can produce equal brake force should be tracked. Under this control mode based only on closed-loop clamping force tracking, once the vehicle’s running state parameters (any one of mass, slope, or even friction coefficient between braking pads and disc) change, the vehicle’s deceleration curve will inevitably incur a random fluctuation, which seriously destroys the vehicle’s braking smoothness. It is also a problem that must be solved for automatic and accurate motion control of autonomous electric mine vehicles. 

**Figure 4:**
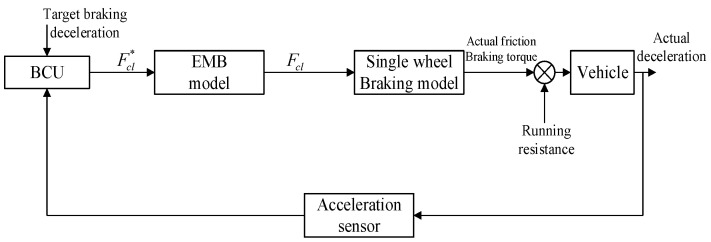
Schematic diagram of constant deceleration control mode.

As shown in Figure 4, the BCU of the constant deceleration control system in our paper directly receives the measured deceleration value from a specific acceleration sensor to construct a deceleration tracking closed-loop controller, whose output is just the input of the clamping force tracking loop. There are many factors, such as friction force between disc and pads, friction between tire and road, vehicle load, road slope, and so on, affecting braking deceleration. Considering the difficulty of accurately identifying multi-parameters, the proposed constant deceleration algorithm is based on fuzzy neural network, which does not rely on specific modeling of the controlled object. With fuzzy neural network to adaptively adjust PID parameters, this deceleration controller can keep actual deceleration at a nearly constant value. The proposed fuzzy neural network algorithm is also written as a C program embedded into the micro-processor of this BCU to implement a real braking control apparatus, which is introduced in detail in Section 3 and Section 4. 

## 3. Closed-Loop Control Strategies of Constant Deceleration Based on Fuzzy Neural-Network PID

### 3.1. Fuzzy Neural Network-Based PID Deceleration Controller Design

**Figure 5:**
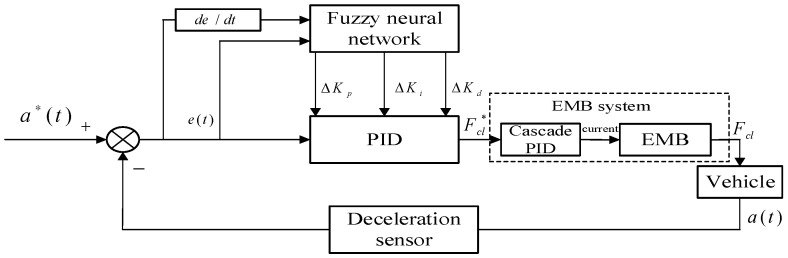
Schematic diagram of Fuzzy Neural Network PID controller.

Although there have been many new industrial control algorithms, the majority of electromechanical control systems still utilize the classical structure of PID. However, the single PID controller has the inherent defect of fixed parameters. In particular, these working parameters are not always proper for target status parameter tracking because the controlled object such as an operating vehicle must have variable parameters of the vehicle model. To solve this problem, this paper designed a Fuzzy Neural Network PID controller, as shown in Figure 5, which utilizes advantages of adaptive tuning parameters of the two algorithms and calculates the adjustment quantity of three PID gain parameters $K_{p},K_{i},K_{d}$ with input variables of deceleration deviation and its rate of change to further carry out the online self-adaptive control. 

The equation of the PID controller is described as follows:

(9) $$F_{cl}^{*}=K_{p}e(t)+K_{i}\int_{0}^{t}e(t)dt+K_{d}\frac{de(t)}{dt}$$

where the output of the PID controller is the target clamping force $F_{cl}^{*}$ and the relations between the equation of the PID controller and the output of the fuzzy neural network $∆K_{p},\;∆K_{i},\;∆K_{d}$ is as follows:

(10) $$\left\{\begin{array}{l} K_{p}=K_{p0}+\Delta K_{p}K_{up}\\ K_{i}=K_{i0}+\Delta K_{i}K_{ui}\\ K_{d}=K_{d0}+\Delta K_{d}K_{ud} \end{array}\right.$$

where $K_{p0},\;K_{i0},\;K_{d0}$ are the initial values of the PID controller or the gain parameter values of the previous sample time step, and $K_{up},K_{ui}$ and $K_{ud}$ are the scale factors for $∆K_{p},\;∆K_{i},\;∆K_{d}$, respectively. 

In terms of force control of the EMB actuator, a cascaded PID controller with two basic PID controllers is designed to track the target clamping force decided by the proposed FNN-PID controller in the form of a closed loop. Input and output signals of the front first PID controller for clamping force control are $F_{cl}^{*}$ and the target speed of the motor, and the input and output of the subsequent PID controller are the target speed of the motor and target current, as shown in Figure 5. The total expression of the block diagram “Cascaded PID” is expressed as follows:

(11) $$u_{o}(s)=u_{i}(s)(K_{p1}+\frac{K_{i1}}{s})(K_{p2}+\frac{K_{i2}}{s})$$

where $K_{p1},\;K_{i1}$ are the proportional and integral gain parameters, respectively, and $K_{p2}$ $K_{i2}$ are the proportional and integral gain parameters, respectively. 

The target current is sent into the electric motor model of the EMB actuator. And the actuator outputs are the response of the clamping force. 

**Figure 6:**
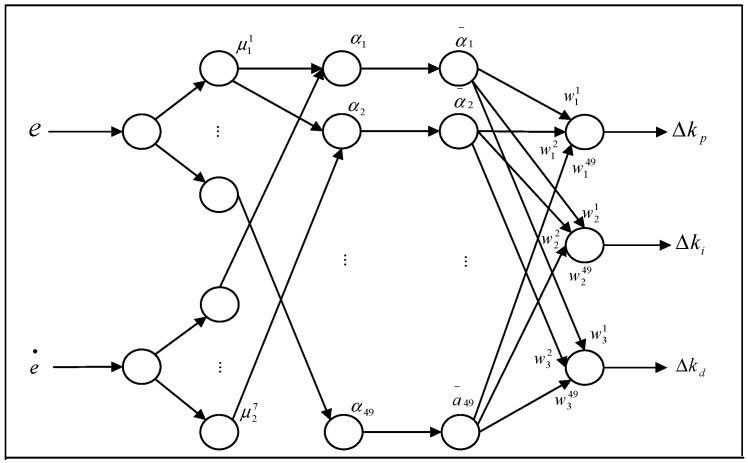
Fuzzy neural network applied to the constant deceleration control for UETRV.

A neural network parameter regulator based on fuzzy optimization for the proposed constant deceleration control system is shown in Figure 6. The controller is a five-layer Deep Neural Network (DNN). The input layer has two nodes, which receive the deceleration error and its change rate, respectively. The middle layers are the fuzzy membership function layer, fuzzy reasoning layer, and normalized output layer. The output layer has three nodes, which are responsible for outputting $∆K_{p},\;∆K_{i},\;∆K_{d}$. 

The equation describing the input layer is as shown below:

(12) $$\begin{array}{l} Input:I_{i}^{1}=x_{i}\\ Output:O_{ij}^{1}=x_{ij}=x_{i} \end{array}$$

where *i* = 1, 2; *j* = 1, 2, …, 7. 

The fuzzy set adopted has seven fuzzy variable expressions, and the role of the second layer is to calculate the membership degree of the input quantity related to every variable in the fuzzy set based on the Gauss function, which realizes the fuzzy expression of the two input quantities and further supports fuzzy reasoning in the next layer.

(13) $$\begin{array}{l} Input:I_{ij}^{2}=x_{ij}=x_{i}\\ Output:O_{ij}^{2}=e^{-\frac{{\left(x_{i}-c_{ij}\right)}^{2}}{\sigma_{ij}^{2}}}=\mu_{i}^{j}(x_{i}) \end{array}$$

where *i* = 1, 2; *j* = 1, 2, …, 7. $\mu_{i}^{j}$ is the membership function of the jth fuzzy subset of the ith input. $c_{ij}$ and $\sigma_{ij}$ are the central value and width of $\mu_{i}^{j}$. 

There are 49 nodes in the fuzzy reasoning layer, which have a point-to-point mapping relationship to the 49 fuzzy rules in the rule base and calculate the adaptability degree of fuzzy language variables to each rule based on the input variables of the previous layer so as to complete the output of the reasoning result.

(14) $$\begin{array}{l} Input:I_{k}^{3}=O_{1i}^{2}O_{2j}^{2}=\mu_{1}^{i}(x_{1})\mu_{2}^{j}(x_{2})\\ Output:O_{k}^{3}=I_{k}^{3}=\alpha_{k} \end{array}$$

where *i* = 1, 2, …, 7; *j* = 1, 2, …, 7; *k* = 1, 2, …, 49. 

The number of nodes in the last layer of the hidden layer is equal to the number of nodes in the previous layer, which functions as the tool to achieve the unified expression of reasoning results.

(15) $$\begin{array}{l} Input:I_{i}^{4}=O_{i}^{3}=\alpha_{i}\\ Output:O_{j}^{4}=\frac{\alpha_{j}}{\sum_{i=1}^{49}\alpha_{i}}=\bar{\alpha_{j}} \end{array}$$

where *i* = 1, 2, …, 7; *j* = 1, 2, …, 7; *k* = 1, 2, …, 49. 

The function of the output layer is to defuzzify the result of fuzzy reasoning, and then calculate and output the dynamically variable gain parameter adjustment values $∆K_{p},\;∆K_{i},\;∆K_{d}$.

(16) $$\begin{array}{l} Input:I_{i}^{5}=\omega_{i}^{j}{\overset{\_\_}{\alpha }}_{j}\\ Output:O_{i}^{5}=I_{i}^{5} \end{array}$$

where *i* = 1, 2, 3; *j* = 1, 2, …, 7. $\omega_{i}^{j}$ is the weight coefficient during defuzzification. 

### 3.2. Training of the Neural Network

The second-order error is adopted as an evaluation criterion, and the back-propagation method and one-order gradient optimization are used to train the center value $c_{ij}$ and width value $\sigma_{ij}$ of the fuzzy control, as well as the weights $\omega_{i}^{j}$ between the nodes connected with each other in the neural network, and calculate $\partial E/\partial w_{i}^{j}$, $\partial E/\partial c_{i}^{j}$, and $\partial E/\partial \sigma_{i}^{j}$

(17) $$E(n)=\frac{1}{2}\sum_{i=1}^{n}(r_{i}-y_{i}{)}^{2}$$

where $y_{i}$ is the actual output of the fuzzy neural network, and $r_{i}$ is the expected one. 

The training equations of $\omega_{i}^{j}$ are

(18) $$\frac{\partial E}{\partial \omega_{i}^{j}}=-(r_{i}-y_{i}){\overset{\_\_}{\alpha }}_{j}$$

(19) $$\omega_{i}^{j}(k+1)=\omega_{i}^{j}(k)-\beta \frac{\partial E}{\partial \omega_{i}^{j}}(i=1,2,3;j=1,2,\cdots ,49)$$

The training equations of width $\sigma_{ij}$ and center $c_{ij}$ are as follows:

(20) $$\frac{\partial E}{\partial c_{ij}}=-\delta_{ij}^{\left(2\right)}\frac{2(x_{i}-c_{ij})}{\sigma_{ij}^{2}}$$

(21) $$\frac{\partial E}{\partial \sigma_{ij}}=-\delta_{ij}^{\left(2\right)}\frac{2{\left(x_{i}-c_{ij}\right)}^{2}}{\sigma_{ij}^{3}}$$

(22) $$c_{ij}(k+1)=c_{ij}(k)-\beta \frac{\partial E}{\partial c_{ij}}(i=1,2;j=1,2,\cdots ,7)$$

(23) $$\sigma_{ij}(k+1)=c_{ij}(k)-\beta \frac{\partial E}{\partial \sigma_{ij}}(i=1,2;j=1,2,\cdots ,7)$$

in which learning rate β>0.

Through simulation of the existing fuzzy PID controller under different target deceleration inputs, the training sample set is generated, and the mentioned parameters of the fuzzy neural network are trained in MATLAB V.2016. 

## 4. Simulated Results and Discussion

### 4.1. Simulation Platform

In order to verify the effectiveness of the controller, this section will construct a simulation model by combining our designed control algorithm and EMB actuator model in Matlab/simulink. According to the design concept of the controller, the controller will be verified from the two aspects of braking force control and deceleration control. 

**Figure 7:**
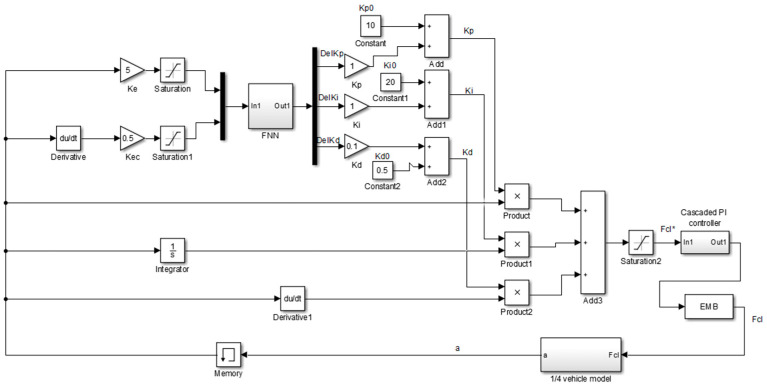
Simulink model of the Fuzzy Neural Network PID.

As depicted in Figure 7, the result of the fuzzy neural network training is the initial PID gain values $K_{p0},\;K_{i0},\;K_{d0}$, as well as the parameters of the fuzzy neural network (FNN). 

### 4.2. Simulation Results and Discussion

**Figure 8:**
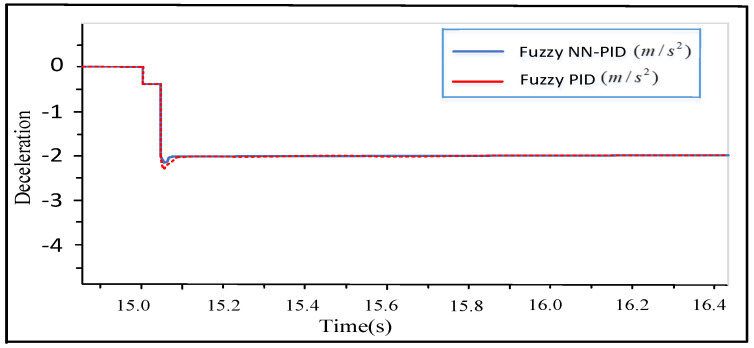
Simulation result of the constant deceleration controller.

For UETRV, there are various working conditions, such as light-load driving, heavy-load driving, driving uphill, and driving downhill. Here, the condition of driving downhill with light load is simulated, and the deceleration reference is $-2\;m/s^{2}$. Compared with the basic Fuzzy PID applied for constant deceleration control [34], FNN-PID can rapidly track the given deceleration and has better performance of transient response (a smaller overshoot), as shown in Figure 8. 

## 5. Experimental Test Results and Discussion

In order to verify the effectiveness of the constant deceleration control, a special deceleration sensor is selected and installed in an experimental electric mine vehicle. The vehicle was subjected to constant deceleration tests on a dedicated test surface simulating the roadway surface. 

### 5.1. Deceleration Sensor Arrangement

**Figure 9:**
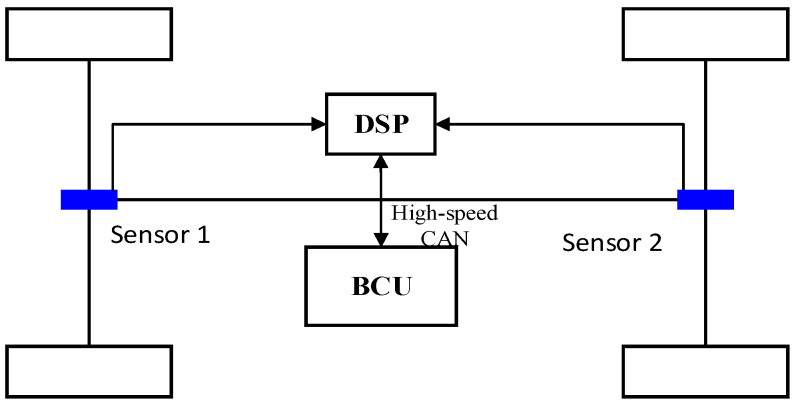
Schematic diagram of installation position of deceleration sensor.

Two similar single-axle deceleration sensors are installed in the front and rear of the test vehicle, respectively, along the longitudinal mid-line of the vehicle in the explosion-proof shell of the braking control unit, as shown in Figure 9.

### 5.2. Experimental Test Result and Discussion

**Figure 10:**
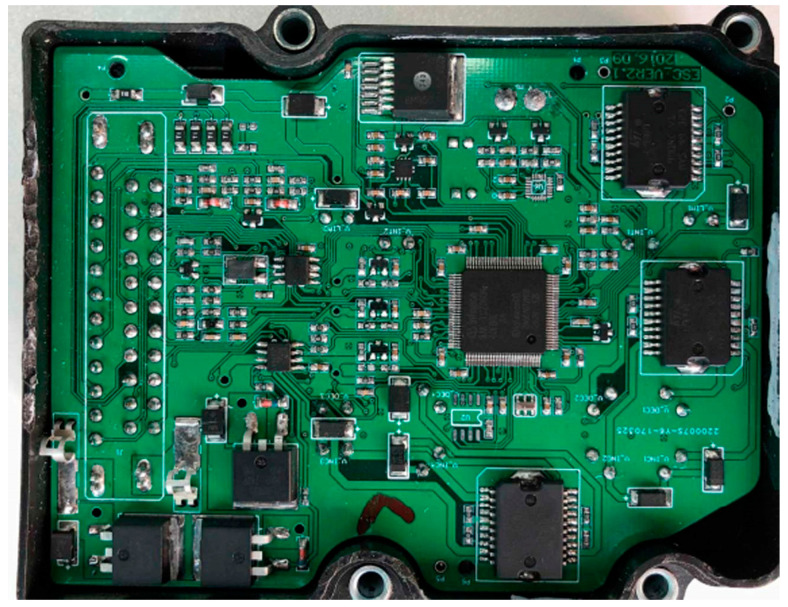
The tested circuit board of this proposed BCU.

Previous simulations have illustrated the feasibility of this constant deceleration controller. Then, an actual braking control unit (BCU) is built, as shown in Figure 10, which is then tested on a real UTRV in a mine. 

Under normal braking conditions, the target deceleration curve of the autonomous driving electric mine vehicle is usually in the form of piecewise uniform deceleration, which is equivalent to a continuous ladder input signal, and the actual movement effect is finally realized by the braking actuator. 

**Figure 11:**
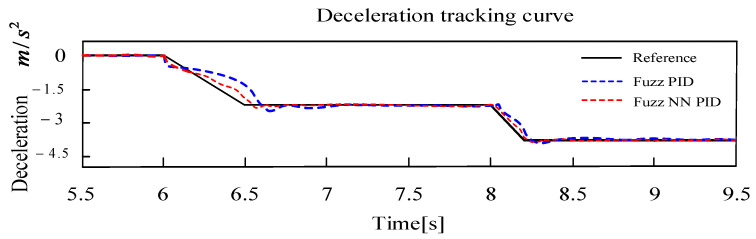
Deceleration tracking curve with two expected values.

**Figure 12:**
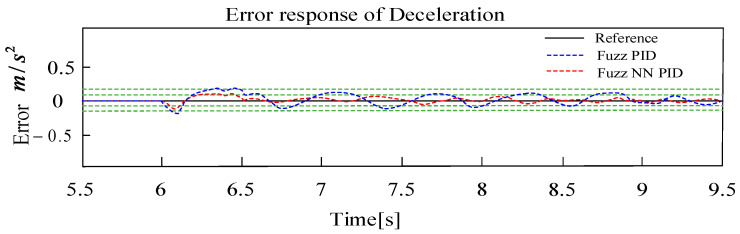
Response of deceleration tracking error.

In Figure 11, the test vehicle is accelerated to 36 km/h and driven at a constant speed for 3s (maximum speed of 35 km/h for the mine electric rubber-tired vehicle), the target deceleration $-2.5\;m/s^{2}$ is set at t = 6 s, and the deceleration target value is switched to $-4\;m/s^{2}\;$ at t = 8 s. Results show that this controller can track continuous and multi-level target deceleration values quickly, which can also restrict the steady-state error within $\pm 0.1\;m/s^{2}$ with an integral square error of 1.8%. The response of the deceleration error can be seen in Figure 12. Obviously, the deceleration error based on the proposed FNN-PID controller is smaller than the one based on basic Fuzzy PID control. Because the dead-time characteristic of the EMB driver is smaller than that of EHB, the transient tracking performance of deceleration is better. 

**Figure 13:**
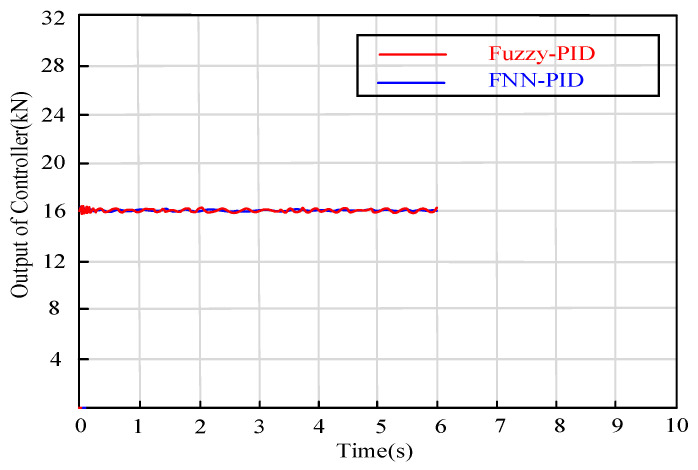
Output curve of controller based on FNN-PID algorithm.

As depicted in Figure 13, the direct output of the proposed fuzzy neural network constant deceleration controller is the target clamping force $F_{cl}^{*}$ of the actuator, which aims to make the automated vehicle keep a constant and smooth deceleration state. As you can see, although the deceleration is a smooth straight line, the clamping force is dynamically adjusted because during the system setup time, the clamping force must be dynamically adjusted to make the system complete the transition process as soon as possible. In the process of a steady state, the controller needs to adjust the output of the controller dynamically because of the dynamic variation of the parameters, such as the friction coefficient of the tire and the friction coefficient of the brake pad, so as to promote the smoothness of the deceleration output; this shows the adaptive ability of the controller to the parameter uncertainty. 

In addition, this proposed constant deceleration controller based on FNN-PID also has the advantage of dealing with the condition of variable load, which is very practically meaningful when the automated electric mine vehicle drives automatically. 

**Figure 14:**
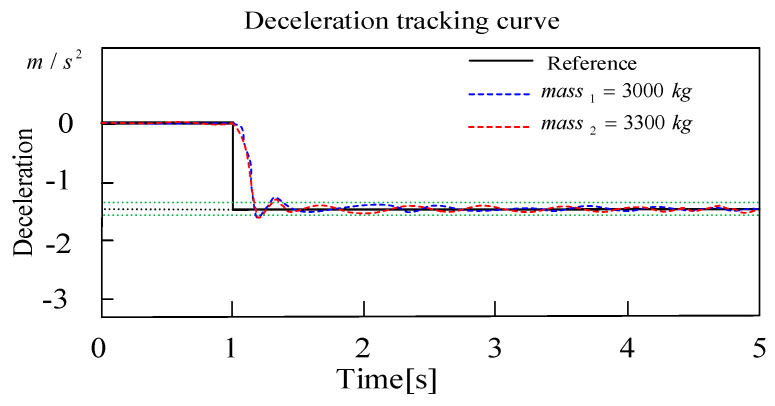
Deceleration tracking curve with two different load levels.

In Figure 14, when the target deceleration is set at $-1.5\;m/s^{2}$, working condition (a) is ${mass}_{1}=3000\;kg$, and condition (b) is ${mass}_{b}=3300\;kg$. Obviously, the controller recalculates the proper target clamping force of the EMB actuator to match the new vehicle load. The two deceleration curves have a similar trajectory, with response times both within 0.28 s and deceleration errors within $\pm 0.1\;m/s^{2}$. It is enough to illustrate its ability to eliminate vehicle parameter uncertainty. 

## 6. Conclusions

This research introduces a fuzzy neural network-based closed-loop deceleration controller using EMB. Because the EMB can present a small effect of the actuator dead-zone, the proposed constant deceleration controller using EMB has better transient response performance. By automatically adapting the clamping force of EMB based on deceleration information feedback, the system maintains a stable deceleration at the desired value. To ensure accuracy in monitoring deceleration of UETRV and reduce computation burden, a specialized deceleration sensor is applied rather than adopting differential of the wheel speed. Simulation results show that the actual deceleration can be maintained at the normal target deceleration value of $-2.5\;m/s^{2}$ and $-4\;m/s^{2}$ with establishment time of 0.28 s and deviation of $\pm 0.1\;m/s^{2}$, which is better than the basic Fuzzy-PID. Experimental tests are conducted on a tested UETVR to evaluate the system’s performance of response and eliminating uncertainty of vehicle mass. The test results demonstrated that the proposed method is effective in achieving a more precise and smooth deceleration tracking performance with variable vehicle mass, which can help to prevent the risk of wheel sliding. Furthermore, the implementation of this algorithm did not cause any time delay of the BCU operation.

In the future, this work aims to consider the estimation of the dynamic friction coefficient between the braking disc and pad. Because the instantaneous friction coefficient undergoes significant change, especially during short-time braking or the initial stage of a continuous braking, the utilization of the instantaneous friction coefficient can significantly improve deceleration control performance and accurate stopping distance during emergency braking, which is very significant for the safety and precision of autonomous mine vehicles. 

## Data Availability

The data that support the findings of this study are available from the corresponding author upon reasonable request.
